# Diagnosis of pneumonia and malaria in Nigerian hospitals: A prospective cohort study

**DOI:** 10.1002/ppul.24691

**Published:** 2020-02-19

**Authors:** Hamish Graham, Ayobami A. Bakare, Adejumoke I. Ayede, Oladapo B. Oyewole, Amy Gray, Eleanor Neal, Shamim A. Qazi, Trevor Duke, Adegoke G. Falade

**Affiliations:** ^1^ Centre for International Child Health, MCRI, Royal Children's Hospital University of Melbourne Parkville Victoria Australia; ^2^ Department of Paediatrics University College Hospital Ibadan Nigeria; ^3^ Department of Community Medicine University College Hospital Ibadan Nigeria; ^4^ Department of Paediatrics University of Ibadan Ibadan Nigeria; ^5^ Infection & Immunity, MCRI Royal Children's Hospital Parkville Victoria Australia; ^6^ Department of Maternal, Newborn, Child and Adolescent Health World Health Organization Geneva Switzerland

**Keywords:** Africa, child, diagnosis, infections: pneumonia, TB, viral, International Health, malaria, Nigeria

## Abstract

**Background:**

Pneumonia and malaria are the leading causes of global childhood mortality. We describe the clinical presentation of children diagnosed with pneumonia and/or malaria, and identify possible missed cases and diagnostic predictors.

**Methods:**

Prospective cohort study involving children (aged 28 days to 15 years) admitted to 12 secondary‐level hospitals in south‐west Nigeria, from November 2015 to October 2017. We described children diagnosed with malaria and/or pneumonia on admission and identified potential missed cases using WHO criteria. We used logistic regression models to identify associations between clinical features and severe pneumonia and malaria diagnoses.

**Results:**

Of 16 432 admitted children, 16 184 (98.5%) had adequate data for analysis. Two‐thirds (10 561, 65.4%) of children were diagnosed with malaria and/or pneumonia by the admitting doctor; 31.5% (567/1799) of those with pneumonia were also diagnosed with malaria. Of 1345 (8.3%) children who met WHO severe pneumonia criteria, 557 (41.4%) lacked a pneumonia diagnosis. Compared with “potential missed” diagnoses of severe pneumonia, children with “detected” severe pneumonia were more likely to receive antibiotics (odds ratio [OR], 4.03; 2.63‐6.16, *P* < .001), and less likely to die (OR, 0.72; 0.51‐1.02, *P* = .067). Of 2299 (14.2%) children who met WHO severe malaria criteria, 365 (15.9%) lacked a malaria diagnosis. Compared with “potential missed” diagnoses of severe malaria, children with “detected” severe malaria were less likely to die (OR, 0.59; 0.38‐0.91, *P* = 0.017), with no observed difference in antimalarial administration (OR, 0.29; 0.87‐1.93, *P* = .374). We identified predictors of severe pneumonia and malaria diagnosis.

**Conclusion:**

Pneumonia should be considered in all severely unwell children with respiratory signs, regardless of treatment for malaria or other conditions.

## 1 INTRODUCTION

Pneumonia and malaria are the leading causes of global mortality in children under 5 years.^1^ In 2017, pneumonia and malaria accounted for almost one‐third of deaths in children under 5 years (excluding the neonatal period).^1^ The majority of pneumonia and malaria cases and deaths occur in low‐ and middle‐income countries, with particularly high burden in sub‐Saharan Africa.^1^, ^2^ In 2017, the WHO Africa region recorded 426 000 pneumonia deaths and 257 000 malaria deaths among children under 5 years of age.^1^ This accounted for 53% of global pneumonia deaths, and 98% of global malaria deaths, for children under 5 years of age during that year.^1^

World Health Organisation (WHO) guidelines recommend a case management approach to the diagnosis and management of pneumonia and malaria.^3^, ^4^ However, the clinical presentation of pneumonia and malaria can be similar, involving fever, fast breathing, and other signs of severe illness such as altered conscious state.^4^ Previous reports have highlighted the risks of under and overdiagnosis of pneumonia and malaria, recommending that, in malaria‐endemic regions, children with a positive malaria test and respiratory signs who require hospitalization should be treated for both pneumonia and malaria.^5^, ^6^ However, we have few data on the clinical overlap of pneumonia and malaria syndromes, or current diagnostic practices, among hospitalized children in high‐burden countries such as Nigeria.^5^, ^6^, ^7^

Nigeria is a populous lower‐middle‐income country in west Africa with high child and neonatal mortality (2017: under‐five mortality 100, neonatal mortality 33, per 1000 live births).^8^ Pneumonia and malaria are the major causes of child deaths, accounting for 18% and 14% of deaths, respectively.^8^ Recent estimates show that Nigeria contributed one‐quarter of under‐five malaria deaths and one‐sixth of under‐five pneumonia deaths globally in 2017.^1^

The aim of this study was to describe the clinical presentation of children diagnosed with pneumonia and/or malaria at secondary‐level hospitals in Nigeria and to explore clinical decision making at the point of admission. Our exploratory analysis identified potential missed cases of severe pneumonia and severe malaria, explored predictors of “correct” pneumonia and malaria admission diagnoses, and considered reasons for clinical overlap and alternative diagnoses in malaria and pneumonia syndromes.

## 2 METHODS

### 2.1 Design

We conducted a prospective cohort study nested within a stepped‐wedge trial evaluating pulse oximetry and oxygen systems in 12 Nigerian hospitals.^9^ The study design has been described previously.^9^ In brief, it involved (a) baseline needs assessment and retrospective clinical review, (b) introduction of pulse oximetry to all participating hospitals (November 2015), and (c) stepped introduction of a comprehensive oxygen system, with hospitals randomized to receive the intervention between March 2016 and March 2017.^9^ We have previously reported results from our needs assessment,^10^, ^11^ process evaluation,^12^, ^13^ and impact evaluation.^14^ This paper reports an exploratory secondary analysis using prospective data from November 2015 to October 2017 extracted from clinical records.

### 2.2 Participants

We conducted this study in southwest Nigeria, recruiting 12 small‐ to medium‐sized hospitals that were representative of secondary healthcare facilities that admit children.^9^ We included government (n = 7) and mission (n = 5) hospitals of varying capacity (Table 1, more details in Appendix 1). All hospitals were low‐altitude (elevation 50‐500 m above sea level) and in malaria‐endemic regions. Pediatric care was provided by junior doctors and generalist nurses under the supervision of family medicine physicians and/or pediatricians (Table 1). Patients requiring additional medical services were referred to larger secondary‐ or tertiary‐level hospitals.

**Table 1:** Hospital characteristics and admission details of 12 secondary‐level hospitals in southwest Nigeria, during the 24‐mo study period (November 2015 to October 2017)

	H1	H2	H3	H4	H5	H6	H7	H8	H9	H10	H11	H12
Hospital characteristics												
Hospital type	Mission	Mission	State	State	State	Mission	State	State	State	Mission	Mission	State
Pediatric beds	70	32	25	36	60	20	48	46	13	63	14	36
child + neonatal	(40 + 30)	(20 + 12)	(21 + 4)	(16 + 20)	(44 + 16)	(15 + 5)	(20 + 28)	(22 + 24)	(9 + 4)	(38 + 25)	(12 + 2)	(26 + 10)
Access to Pediatrician	Yes^a^	No^b^	Yes	Yes	Yes	Yes	Yes	No	No^b^	Yes^a^	Yes^a^	No^b^
Doctors in hospital	4	4	2	11	17	5	16	12	7	6	6	7
Nurses in child/newborn wards	18	7	16	33	62	9	26	31	11	18	4	26
Baseline oxygen in pediatric area^c^												
Oxygen cylinders	No	No	No	Yes	Yes	No	Yes	Yes	No	Yes	No	No
Oxygen concentrators	Faulty	No	Faulty	Faulty	No	Yes	Faulty	No	No	Faulty	Faulty	No
Improved oxygen access^c^	Mar 2016	Mar 2016	Mar 2016	Jul 2016	Jul 2016	Jul 2016	Nov 2016	Nov 2016	Nov 2016	Mar 2017	Mar 2017	Mar 2017
Pulse oximeters^c^	Yes	Yes	Yes	Yes	Yes	Yes	Yes	Yes	Yes	Yes	Yes	Yes
Participants (total)	1837	574	1520	1616	1769	533	2754	1295	479	2123	780	904
Infants 28 d to 1 y	505	176	324	445	599	97	1055	371	114	528	131	186
Children 1‐4 y	884	259	762	784	790	288	1624	569	231	979	312	449
Children 5‐14 y	433	138	429	378	342	143	74	338	133	616	333	267
Age missing	15	1	5	9	38	5	1	17	1	0	4	2
Sex, female (%)	43.9	45.1	46.5	42.6	41.8	42.0	44.7	43.6	44.8	43.7	42.8	40.7
Median age, months (IQR)	23 (11‐57)	21 (9‐54)	30 (12‐60)	22 (11‐54)	20 (8‐49)	31 (15‐62)	14 (8‐24)	24 (11‐62)	27 (12‐60)	24 (12‐60)	46 (15‐97)	24 (12‐60)
Median length of stay, days (IQR)	3 (2‐5)	3 (2‐5)	3 (2‐4)	3 (2‐5)	7 (7‐9)	3 (2‐4)	3 (2‐4)	6 (4‐7)	2 (1‐2)	4 (3‐5)	2 (1‐3)	3 (2‐4)
Deaths	88	36	114	66	57	21	42	65	17	83	27	26
Case fatality rate (%)	4.8	6.3	7.5	4.1	3.2	4.0	1.5	5.0	3.6	3.9	3.5	2.9

*Note*: Child 29 d to 15 y. IQR, interquartile range, 25^th^‐75^th^ centiles. Additional detail from the preintervention facility assessment is available in Web Appendix 1.

Abbreviation: LOS, length of stay.

^a^ Family Medicine Consultant.

^b^ Part‐time.

^c^ All hospitals received pulse oximeters in October/November 2015 and an improved oxygen system between March 2016 and March 2017.

We included all children aged 28 days to 15 years who were admitted to participating hospitals during the study period (Table 1).

### 2.3 Materials and procedures

Trained research nurses used standardized data collection forms to gather data from clinical records immediately following patient discharge. Documented data included participant demographics, signs and symptoms on admission, admission diagnoses, and clinical care practices. Admission documentation was typically performed by junior doctors (medical admission note) and generalist nurses (nursing observations and nursing admission note). Admitting doctors were typically junior doctors who did not have pediatric specialty training, who performed their pediatric clinical duties while also covering other wards, and who would complete admission documentation at the point of admission (outpatient or emergency department) or on the pediatric ward. Admitting nurses were typically generalist nurses or nursing assistants who were based on the pediatric ward and who would complete admission documentation when the child arrived on the ward.

As part of our broader pulse oximetry and oxygen intervention,^9^ we trained doctors and nurses on pulse oximetry and oxygen therapy, in accordance with WHO guidelines.^15^ We did not provide doctors or nurses with additional training or standardization of diagnostic, treatment, or documentation practices. We did not provide any pneumonia‐ or malaria‐specific training or guidance.

### 2.4 Data analysis

Trained data entry clerks used EpiData 3.1^16^ to double‐enter data from data collection forms following standard data management procedures. We performed data cleaning and analysis using Stata 15.1.^17^

We identified children diagnosed with pneumonia or malaria on admission, permitting multiple diagnoses. We accepted all diagnostic terms relating to lower respiratory tract infection for pneumonia (including bronchiolitis) and similarly for malaria. We categorized participant diagnoses as “malaria,” “malaria + pneumonia,” “pneumonia,” or “neither malaria nor pneumonia.” For each category, we summarized demographic and clinical symptoms and signs using descriptive statistics and tests of difference. We summarized categorical variables by number and percentages. We summarized continuous variables by mean and standard deviation (SD) if normally distributed, and by median and interquartile range (25th and 75th centiles) if non‐normally distributed. We used *χ* ^2^ test to evaluate differences between categorical dependent and independent variables, *t* test for dichotomous independent variables on a continuous outcome, and analysis of variance for multiple categorical independent variables on a continuous outcome.

Using WHO case definitions (Box 1), we identified the number of children who met clinical criteria for severe pneumonia or severe malaria diagnosis^4^ in each of the admission diagnosis subgroups. These WHO case definitions are not “gold standard” classifications for severe pneumonia or malaria. Rather, they are clinical syndromes that are intended to guide the assessment, diagnosis, and treatment of severely ill children with conditions that are major causes of childhood mortality.^4^ As described in the *WHO Pocketbook of Hospital Care for Children*, these WHO clinical case definitions are intended for use by generalist doctors and nurses working at first‐referral level health hospitals with limited diagnostic capacity. *WHO Pocketbook* diagnostic and treatment advice is consistent with Nigeria's *Standard Treatment Guidelines* (but provides additional specific advice for the pediatric population)^18^ and WHO primary care guidelines (integrated management of childhood illness).^4^

Box 1 **Clinical case definitions for severe pneumonia and severe malaria, derived from the WHO Pocketbook of Hospital Care for Children.^4^**

Severe pneumonia

Cough or Difficult breathing AND at least one of the following:

- severe respiratory distress;
- hypoxemia (SpO_2_<90%);
- unable to breastfeed or drink adequately;
- decreased conscious state;
- seizures.

Severe malaria

Fever (or history of fever) AND positive malaria test (blood film or rapid diagnostic test) AND at least one of the following:

- severe anemia;
- decreased conscious state;
- seizures.

Both the admission diagnosis and the clinical case definition classifications were derived from the admitting doctor and nurse's documentation at the time of admission. If a clinical sign was not recorded, we assumed that it was not present.

By focusing on admission diagnoses and clinical case definitions, we were able to explore real‐life diagnostic and management practices in the critical first hours of a severely ill child presenting to hospital. We categorized those meeting WHO criteria for severe pneumonia as “detected” or “potential missed” depending on whether they had a corresponding admission diagnosis of pneumonia. We categorized those meeting WHO criteria for malaria similarly.

To understand diagnostic decision making, we compared “detected” vs “potential missed” severe pneumonia subgroups. We built univariate logistic regression models to look for associations between presenting demographic characteristics, clinical signs, investigations, treatment, and clinical outcomes, with a “detected” pneumonia and malaria diagnosis. We then used a mixed effects logistic regression model to evaluate the relative contribution of demographic characteristics, clinical signs, and investigations to pneumonia and malaria diagnostic status (“detected” vs “potential missed”). We selected variables for inclusion in the multivariate model using backward and forward stepwise regression. We used random effects to adjust for clustering at the hospital level.

We report odds ratios (ORs), adjusted odds ratios (aORs), and 95% confidence intervals (95% CI) using complete case analysis approach (ie, dropping missing data from analysis).^19^

## 3 RESULTS

**Figure 1:**
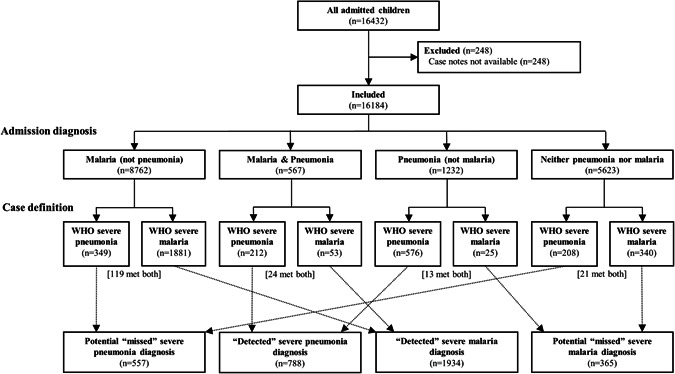
Participant flow chart

In the 24‐month study period (November 2015 to October 2017), 16 432 children were admitted to participating hospitals. Case notes were available for 16 184 participants (98.5%) (Figure 1).

### 3.1 Admission diagnoses

Two‐thirds (10 561, 65.4%) of admitted children had an admission diagnosis of malaria and/or pneumonia (Figure 1). Of these, 9329 (88.3%) were diagnosed with malaria and 1799 (17.0%) were diagnosed with pneumonia, including 567 (5.4%) who were diagnosed with both malaria and pneumonia. One‐third of those diagnosed with pneumonia were also diagnosed with malaria (567/1799, 31.5%).

### 3.2 Characteristics of children diagnosed with pneumonia and malaria

Children diagnosed with pneumonia were younger than those with malaria or other diagnoses (median age 9.8 vs 26 and 18 months, *P* < .001) (Table 2). Most children presented with a history of fever (13 144, 80.0%), while children diagnosed with malaria tended to have higher admission temperature than those without (37.8 vs 37.4°C, *P* < .001). Children diagnosed with pneumonia were more likely to have cough or difficult breathing (86.4% vs 19.6%, *P* < .001) and severe respiratory distress (39.8% vs 4.7%, *P* < .001) than those diagnosed with malaria. They also tended to have a higher respiratory rate (53.4 vs 37.2, *P* < .001) and were more likely to have hypoxemia (31.2% vs 7.3%, *P* < .001). Children diagnosed with malaria were more likely to have seizures (16.9% vs 3.3%, *P* < .001), altered conscious state (7.9% vs 3.0%, *P* < .001), and signs of severe dehydration (2.8% vs 0.5%, *P* < .001) than those diagnosed with pneumonia.

**Table 2:** Participant characteristics, comparing those with admission diagnoses of malaria, malaria‐pneumonia, pneumonia, or neither malaria nor pneumonia

Characteristic	Malaria (not pneumonia)	Malaria and Pneumonia	Pneumonia (not malaria)	Other (neither malaria nor pneumonia)	*P* ^a^
Participants (total)	8762 (100)	567 (100)	1232 (100)	5623 (100)	
Infants 28 d to 1 y	1777 (20.3)	239 (42.2)	686 (55.7)	1829 (32.5)	<.001
Children 1‐4 y	4750 (54.2)	274 (48.3)	463 (37.6)	2444 (43.5)	<.001
Children 5‐14 y	2185 (24.9)	50 (8.8)	77 (6.3)	1312 (23.3)	<.001
Age unknown (missing)	50 (0.6)	4 (0.7)	6 (0.5)	38 (0.7)	.337
Sex, female (%)	43.7	41.5	44.5	43.5	.694
Median age, months (IQR)	26.0 (12.5‐60.0)	15.0 (7.0‐26.6)	9.8 (3.6‐21.3)	18.4 (9.0‐53.7)	<.001
Admission source: home vs other	8053 (92.6)	494 (88.4)	1114 (91.3)	5178 (92.9)	<.001
Hospital type: government vs other	5356 (61.1)	313 (55.2)	842 (68.3)	3826 (38.0)	<.001
Median LOS, days (IQR)	3 (2‐5)	4 (3.7)	4 (2‐7)	3 (2‐6)	<.001
Documented signs and symptoms					
History of fever	7979 (91.1)	504 (88.9)	868 (70.5)	3793 (67.5)	<.001
History of cough or difficult breathing	1714 (19.6)	460 (81.1)	1064 (86.4)	992 (17.6)	<.001
Severe respiratory distress	412 (4.7)	155 (27.3)	490 (39.8)	251 (4.5)	<.001
Central cyanosis	57 (0.7)	3 (0.5)	11 (0.9)	23 (0.4)	.108
Pallor	3222 (36.8)	130 (22.9)	194 (15.8)	1301 (23.1)	<.001
Jaundice	354 (4.0)	8 (1.4)	14 (1.1)	213 (3.8)	<.001
Diarrhea	999 (11.4)	33 (5.8)	39 (3.2)	989 (17.6)	<.001
Feeding difficulty	1918 (21.9)	107 (18.9)	157 (12.7)	980 (17.4)	<.001
Seizures	1481 (16.9)	55 (9.7)	41 (3.3)	503 (9.0)	<.001
Confusion or lethargy	1131 (12.9)	41 (7.2)	60 (4.9)	578 (10.3)	<.001
Unconscious or barely conscious	692 (7.9)	30 (5.3)	37 (3.0)	267 (4.8)	<.001
Signs of shock	26 (0.3)	0 (0.0)	3 (0.24)	17 (0.3)	.778
Severe dehydration	248 (2.8)	8 (1.4)	6 (0.5)	346 (6.2)	<.001
Mean temperature, Celsius (SD)	37.8 (1.1)	37.8 (1.1)	37.4 (1.0)	37.4 (1.1)	<.001
Mean heart rate, bpm (SD)	131.8 (24.8)	140.6 (25.6)	141.7 (26.3)	129.1 (24.9)	<.001
Mean respiratory rate, bpm (SD)	37.2 (11.7)	49.0 (15.9)	53.4 (17.6)	36.7 (11.6)	<.001
Median SpO_2_, % (IQR)	97 (95‐98)	95 (91‐98)	94 (87‐97)	97 (95‐98)	<.001
Hypoxemia, SpO_2_<90%	501 (7.3)	105 (21.8)	332 (31.2)	359 (8.3)	<.001
Investigations					
PCV performed	8059 (92.0)	523 (92.2)	1036 (84.1)	4683 (83.3)	<.001
Anemia	5047 (62.6)	296 (56.6)	509 (49.1)	2465 (52.6)	<.001
Severe anemia	951 (11.8)	29 (5.5)	11 (1.1)	233 (5.0)	<.001
Mean PCV, % (SD)	26.8 (8.9)	29.3 (7.5)	31.1 (5.8)	29.7 (7.7)	<.001
Malaria test done	7456 (85.1)	496 (87.5)	927 (75.2)	4000 (71.1)	<.001
RDT	3771 (43.0)	252 (44.4)	405 (32.9)	1764 (31.4)	<.001
Microscopy	3723 (42.5)	247 (43.6)	527 (42.8)	2254 (40.1)	.022
Malaria test positive	5002 (67.1)	256 (51.6)	255 (27.5)	1334 (33.4)	<.001
RDT	2307 (61.2)	116 (46.0)	69 (17.0)	407 (23.1)	<.001
Microscopy	2701 (72.6)	141 (57.1)	187 (35.5)	928 (41.2)	<.001
HIV test done	2677 (30.6)	170 (30.0)	337 (27.4)	1648 (29.3)	.089
HIV‐positive	28 (1.1)	5 (2.9)	9 (2.7)	41 (2.5)	<.001
CXR performed	21 (0.2)	83 (14.6)	113 (9.2)	25 (0.4)	<.001
CXR positive^b^	12/13 (92.3)	59/70 (84.3)	81/100 (81.0)	13/19 (68.4)	NA
Case definition diagnoses					
Severe malaria	1881 (21.5)	53 (9.4)	25 (2.0)	340 (6.1)	<.001
Severe pneumonia	349 (4.0)	212 (37.4)	576 (46.8)	208 (3.7)	<.001
Other admission diagnoses					
Diarrhea	1601 (18.3)	45 (7.9)	52 (4.2)	1680 (30.6)	<.001
Sepsis	2902 (33.1)	133 (23.5)	161 (13.1)	1896 (34.5)	<.001
Seizures	841 (9.6)	25 (4.4)	25 (2.0)	403 (7.3)	<.001
Haemoglobinopathy	310 (3.5)	6 (1.1)	11 (0.9)	403 (7.3)	<.001
Meningitis/encephalitis	306 (3.5)	15 (2.7)	10 (0.8)	183 (3.3)	<.001
Severe acute malnutrition	150 (1.7)	7 (1.2)	19 (1.5)	191 (3.5)	<.001
Asthma	19 (0.2)	7 (1.2)	27 (2.2)	55 (1.0)	<.001
Outcome					
Discharged well	7720 (88.1)	483 (85.2)	1014 (82.3)	4707 (83.7)	<.001
Died	269 (3.1)	30 (5.3)	80 (6.5)	263 (4.7)	<.001
Discharged against medical advice	571 (6.5)	35 (6.2)	92 (7.5)	426 (7.5)	.074
Transferred to other facility	170 (2.0)	18 (3.2)	45 (3.7)	209 (3.7)	<.001
Missing data	32 (0.4)	1 (0.2)	1 (0.1)	18 (0.3)	.374

*Note*: Number (%) unless otherwise indicated. Anemia = PCV < 32%. Died = died in hospital or palliated at home. Feeding difficulty, unable to breastfeed or drink adequately. IQR = interquartile range, 25th to 50th centile. Severe anemia, PCV < 15%. Severe dehydration, sluggish capillary refill time (>3 s), sunken eyes, decreased skin turgor. Severe malaria = fever or history of fever AND positive malaria test AND at least one of the following: severe anemia; decreased conscious state; seizures. Severe pneumonia = cough or difficult breathing AND at least one of the following: severe respiratory distress; hypoxemia (SpO_2_ < 90%); unable to breastfeed or drink adequately; decreased conscious state; seizures. Severe respiratory distress = severe chest indrawing, grunting, gasping. Signs of shock = capillary refill < 3 s, weak pulse, low or unmeasurable blood pressure.

Abbreviations: ANOVA, Analysis of variance; PCV, packed cell volume (hematocrit); SD, standard deviation.

^a^ *P* values derived from *χ* ^2^ or Fisher's exact test for categorical variables and *t* test or ANOVA for continuous independent variables.

^b^ CXR denominator restricted to those with results available.

Most children were tested for anemia (14 301, 88.4%) and malaria (12 879, 79.6%) (Table 2). Approximately half the children tested for anemia had low packed cell volume (PCV) (8317 of 14 301, 58.2%), and severe anemia (PCV < 15%) was more common among those diagnosed with malaria than pneumonia (11.8% vs 1.1%, *P* < .001). HIV prevalence was low (<1% of admissions). Chest X‐ray radiography was performed infrequently (242, 1.5%), even among those diagnosed with pneumonia (196 of 1799, 10.9%).

Children diagnosed with pneumonia were more likely to die (6.5% vs 3.1%, *P* < .001) or be transferred to another facility (3.7% vs 2.0%, *P* < .001) compared with those diagnosed with malaria (Table 2).

### 3.3 Severe pneumonia

Of all admitted children, 1345 (8.3%) met the WHO case definition for severe pneumonia (Figure 1, Table 2). Of these, 557 (41.4%) did not have pneumonia as an admission diagnosis, representing potential missed severe pneumonia diagnoses.

Compared with those with potential “missed” severe pneumonia diagnoses, those with “detected” severe pneumonia were younger (*P* < .001), more likely to have severe respiratory distress (aOR 3.87; 95% CI, 2.58‐5.79) and hypoxemia (aOR 2.01; 0.37‐2.96), less likely to have diarrhea (aOR 0.29; 0.14‐0.63), feeding difficulty (aOR 0.59; 0.38‐0.90), seizures (aOR 0.26; 0.15‐0.44), confusion/lethargy (aOR 0.47; 0.27‐0.84), severe dehydration (aOR 0.09; 0.01‐0.53), positive malaria test (aOR 0.49; 0.34‐0.71), or severe anemia (aOR 0.20; 0.09‐0.45), and had lower admission temperature (aOR 0.78; 0.66‐0.92) (Table 3).

**Table 3:** Associations with “detected” severe pneumonia diagnosis and presenting signs, treatments, and outcome

	WHO severe pneumonia		Odds ratio: detected severe pneumonia			
Characteristic	“Potential missed” diagnosis	“Detected” diagnosis	OR (95% CI)	*P* ^a^	aOR (95% CI)^b^	*P*
Participants (total)	557 (100)	788 (100)				
Infants 28 d to 1 y	168 (30.2)	452 (57.4)	Reference	…		
Children 1‐4 y	306 (54.9)	296 (37.6)	0.36 (0.28‐0.46)	<.001		
Children 5‐14 y	81 (14.5)	36 (4.6)	0.17 (0.11‐0.25)	<.001		
Age unknown (missing)	2 (0.4)	4 (0.5)	Not applicable			
Sex, female (%)	44.3	45.3	0.96 (0.77‐1.20)	.957	…	…
Median age, (IQR) mo	19.0 (9.0‐36.0)	9.0 (3.4‐19.0)	0.98 (0.97‐0.98)	<.001	0.98 (0.97‐0.98)	<.001
Admission source: home vs other	497 (89.4)	701 (89.4)	1.00 (0.70‐1.43)	.988	…	…
Hospital type: government vs other	304 (54.6)	481 (61.0)	0.77 (0.62‐0.96)	.018	…	…
Median LOS, (IQR) d	4 (2‐6)	4 (2‐6)	1.05 (1.01‐1.08)	.008	…	…
Documented signs and symptoms						
History of fever	479 (86.0)	585 (74.2)	0.47 (0.35‐0.63)	<.001	…	…
History of cough or difficult breathing	557 (100)	788 (100)	Not applicable			
Severe respiratory distress	240 (43.1)	573 (72.7)	3.52 (2.80‐4.43)	<.001	3.87 (2.58‐5.79)	<.001
Central cyanosis	13 (2.3)	10 (1.3)	0.54 (0.23‐1.24)	.144	…	…
Pallor	262 (47.0)	166 (21.1)	0.30 (0.24‐0.38)	<.001	…	…
Jaundice	27 (4.9)	8 (1.0)	0.20 (0.09‐0.45)	<.001	…	…
Diarrhea	58 (10.4)	29 (3.7)	0.33 (0.21‐0.52)	<.001	0.29 (0.14‐0.63)	.002
Feeding difficulty	194 (34.8)	161 (20.4)	0.48 (0.38‐0.61)	<.001	0.59 (0.38‐0.90)	.015
Seizures	130 (23.3)	50 (6.4)	0.22 (0.16‐0.31)	<.001	0.26 (0.15‐0.44)	<.001
Confusion or lethargy	109 (19.6)	63 (8.0)	0.36 (0.26‐0.50)	<.001	0.47 (0.27‐0.84)	.011
Unconscious or barely conscious	80 (14.4)	34 (4.3)	0.27 (0.18‐0.41)	<.001	…	…
Signs of shock	10 (1.8)	3 (0.4)	0.21 (0.06‐0.76)	.018	…	…
Severe dehydration	24 (4.3)	10 (1.3)	0.29 (0.14‐0.60)	.001	0.09 (0.01‐0.53)	.008
Mean temperature, (SD) °C	37.9 (1.2)	37.6 (1.1)	0.78 (0.70‐0.86)	<.001	0.78 (0.66‐0.92)	.003
Mean heart rate, (SD) bpm	145 (26)	146 (27)	1.00 (1.00‐1.01)	.620	…	…
Mean respiratory rate, (SD) bpm	53 (16)	59 (17)	1.02 (1.02‐1.03)	<.001	…	…
Median SpO_2_, % (IQR)	94 (86‐98)	90 (81‐96)	0.99 (0.98‐0.99)	<.001	…	…
Hypoxemia, SpO_2_ < 90%	149/472 (31.6)	337/698 (48.3)	2.02 (1.58‐2.58)	<.001	2.01 (0.37‐2.96)	<.001
Investigations						
PCV performed	497 (89.2)	682 (86.6)	0.78 (0.55‐1.09)	.142	…	…
Anemia	353 (71.0)	360 (52.8)	0.46 (0.36‐0.58)	<.001	…	…
Severe anemia	89 (17.9)	13 (1.9)	0.09 (0.05‐0.16)	<.001	0.20 (0.09‐0.45)	<.001
Mean PCV, % (SD)	25.0 (9.6)	30.5 (6.2)	1.09 (1.07‐1.11)	<.001	…	…
Malaria test done	410 (73.6)	589 (74.8)	1.06 (0.83‐1.36)	.638	…	…
RDT	220 (39.5)	306 (38.8)	0.97 (0.78‐1.21)	.806	…	…
Microscopy	190 (34.1)	286 (36.3)	1.10 (0.88‐1.38)	.410	…	…
Malaria test positive	252 (61.5)	202 (34.3)	0.33 (0.25‐0.43)	<.001	0.49 (0.34‐0.71)	<.001
RDT	125 (56.8)	76 (24.8)	0.25 (0.17‐0.36)	<.001	…	…
Microscopy	127 (66.8)	127 (44.4)	0.40 (0.27‐0.58)	<.001	…	…
HIV test done	247 (44.3)	257 (32.6)	0.61 (0.49‐0.76)	<.001	…	…
HIV‐positive	7 (2.8)	10 (3.9)	1.39 (0.52‐3.71)	.513	…	…
CXR performed	22 (4.0)	118 (15.0)	4.28 (2.68‐6.85)	<.001	…	…
CXR positive^c^	13/14 (92.9)	82/101 (81.2)	Not applicable			
Other admission diagnoses						
Malaria	349 (64.3)	217 (26.8)	0.31 (0.27‐0.36)	<.001	Not applicable	
Diarrhea	62 (11.4)	26 (3.3)	0.26 (0.17‐0.42)	<.001	Not applicable	
Sepsis	205 (37.8)	103 (13.1)	0.25 (0.19‐0.32)	<.001	Not applicable	
Seizures	66 (12.2)	23 (2.9)	0.22 (0.13‐0.35)	<.001	Not applicable	
Haemoglobinopathy	16 (3.0)	6 (0.8)	0.25 (0.10‐0.65)	.004	Not applicable	
Meningitis/encephalitis	28 (5.2)	8 (1.0)	0.19 (0.09‐0.42)	<.001	Not applicable	
Severe acute malnutrition	24 (4.4)	9 (1.1)	0.25 (0.12‐0.54)	<.001	Not applicable	
Asthma	33 (6.1)	19 (2.4)	0.38 (0.21‐0.68)	.001	Not applicable	
Treatment						
Parenteral antibiotic on day 1	472 (85.4)	751 (95.9)	4.03 (2.63‐6.16)	<.001	Not applicable	
IV Fluids	419 (75.8)	520 (66.4)	0.63 (0.50‐0.81)	<.001	Not applicable	
Outcome						
Discharged well	423 (76.4)	628 (79.9)	1.23 (0.95‐1.60)	.121	Not applicable	
Died	68 (12.3)	72 (9.2)	0.72 (0.51‐1.02)	.067	Not applicable	
Discharged against medical advice	33 (6.0)	57 (7.3)	1.23 (0.79‐1.92)	.352	Not applicable	
Transferred to other facility	30 (5.4)	29 (3.7)	0.67 (0.40‐1.13)	.132	Not applicable	
Missing data	3 (0.5)	2 (0.3)	0.47 (0.08‐2.82)	.409	Not applicable	

*Note*: Number (%) unless otherwise indicated. Anemia = PCV < 32%. Died = died in hospital or palliated at home. Feeding difficulty = unable to breastfeed or drink adequately. IQR = interquartile range, 25th to 50th centile. Severe anemia = PCV < 15%. Severe dehydration = sluggish capillary refill time (>3 s), sunken eyes, decreased skin turgor. Severe pneumonia = cough or difficult breathing AND at least one of the following: severe respiratory distress; hypoxemia (SpO_2_ < 90%); unable to breastfeed or drink adequately; decreased conscious state; seizures. Severe respiratory distress = severe chest indrawing, grunting, gasping. Signs of shock = capillary refill < 3 s, weak pulse, low or unmeasurable blood pressure).

Abbreviations: PCV, packed cell volume (hematocrit); SD, standard deviation.

^a^ *P* values derived from univariate logistic regression.

^b^ Adjusted odds ratio derived from mixed effects logistic regression model that included random effects to adjust for clustering at the hospital level, and used stepwise regression to select variables that were independently associated with ‘detected’ diagnosis at alpha 0.05 level (excluding treatment and outcome variables).

^c^ CXR denominator restricted to those with results available.

Results from univariate logistic regression show that those with “detected” severe pneumonia had greater odds of receiving parenteral antibiotics on day 1 (OR, 4.03; 2.63‐6.16, *P* < .001; 95.9% vs 85.4%) and lower odds of receiving intravenous fluids (OR, 0.63; 0.50‐0.81, *P* < .001; 66.4% vs 75.8%) compared with those with “potential missed” severe pneumonia (Table 3). There was a negative association between mortality and detected severe pneumonia; however, the 95% CI crossed the null value (OR, 0.72; 0.51‐1.02, *P* = .067).

### 3.4 Severe malaria

Of all admitted children, 2299 (14.2%) met the case definition for severe malaria. Of these, 365 (15.9%) were not diagnosed with malaria on admission, representing potential missed severe malaria diagnoses.

Compared with those with potential “missed” severe malaria diagnoses, those with “detected” severe malaria were more likely to have a history of fever (aOR 3.78; 95% CI, 1.98‐7.23) and anemia (aOR 0.40; 0.21‐0.74), and less likely to have a history of cough or difficult breathing (aOR 0.71; 0.52‐0.97), severe respiratory distress (aOR 0.60; 0.38‐0.93), diarrhea (aOR 0.33; 0.21‐0.50), and severe dehydration (aOR 0.40; 0.21‐0.74) (Table 4).

**Table 4:** Associations with “detected” severe malaria diagnosis and presenting signs, treatments, and outcome

	WHO severe malaria		Odds ratio: detected severe malaria diagnosis			
Characteristic	“Potential missed” diagnosis	“Detected” diagnosis	OR (95% CI)	*P* ^a^	aOR (95% CI)^b^	*P*
Participants (total)	365 (100)	1934 (100)				
Infants 28 d to 1 y	85 (23.3)	254 (13.1)	Reference	…		
Children 1‐4 y	189 (51.8)	1235 (63.9)	2.19 (1.64‐2.92)	<.001		
Children 5‐14 y	88 (24.1)	432 (22.3)	1.64 (1.17‐2.30)	.004		
Age unknown (missing)	3 (0.8)	13 (0.7)	Not applicable			
Sex, female (%)	43.7	44.4	0.97 (0.77‐1.22)	.791	…	…
Median age, mo (IQR)	27.3 (12.0‐58.0)	32.0 (18.0‐52.6)	1.00 (1.00‐1.00)	.698	…	…
Admission source: home vs other	332 (91.0)	1695 (88.2)	0.75 (0.51‐1.09)	.134	…	…
Hospital type: government vs other	196 (53.7)	1162 (60.1)	0.77 (0.62‐0.96)	.023	…	…
Median LOS, days (IQR)	4 (2‐5)	4 (2‐5)	1.01 (0.98‐1.04)	.555	…	…
Documented signs and symptoms						
History of fever	342 (93.7)	1906 (98.6)	4.58 (2.61‐8.04)	<.001	3.78 (1.98‐7.23)	<.001
History of cough or difficult breathing	74 (20.3)	335 (17.3)	0.82 (0.62‐1.09)	.177	0.71 (0.52‐0.97)	.034
Severe respiratory distress	35 (9.6)	182 (9.4)	0.98 (0.67‐1.43)	.915	0.60 (0.38‐0.93)	.024
Central cyanosis	0 (0.0)	34 (1.8)	Not applicable			
Pallor	181 (49.6)	1135 (58.7)	1.44 (1.15‐1.81)	.001	…	…
Jaundice	24 (6.6)	130 (6.7)	1.02 (0.65‐1.61)	.918	…	…
Diarrhea	60 (16.4)	115 (6.0)	0.32 (0.23‐0.45)	<.001	0.33 (0.21‐0.50)	<.001
Feeding difficulty	69 (18.9)	478 (24.7)	1.41 (1.06‐1.87)	.017	…	…
Seizures	169 (46.3)	925 (47.8)	1.06 (0.85‐1.33)	.592	…	…
Confusion or lethargy	115 (31.5)	598 (30.9)	0.97 (0.76‐1.24)	.824	…	…
Unconscious or barely conscious	70 (19.2)	444 (23.0)	1.26 (0.95‐1.66)	.113	…	…
Signs of shock	1 (0.3)	15 (0.8)	2.85 (0.37‐21.61)	.312	…	…
Severe dehydration	28 (7.7)	40 (2.1)	0.25 (0.15‐0.42)	<.001	0.40 (0.21‐0.74)	.003
Mean temperature, Celsius (SD)	37.9 (1.1)	37.9 (1.1)	0.99 (0.90‐1.10)	.899	…	…
Mean heart rate, bpm (SD)	137.0 (26.6)	138.5 (26.1)	1.00 (1.00‐1.01)	.305	…	…
Mean respiratory rate, bpm (SD)	39.2 (14.0)	39.9 (12.8)	1.00 (1.00‐1.01)	.322	…	…
Median SpO_2_, IQR (%)	97 (92‐98)	97 (93‐98)	1.01 (1.00‐1.02)	.178	…	…
Hypoxemia, SpO_2_ < 90%	47/295 (15.9)	214/1559 (13.7)	0.84 (0.60‐1.18)	.318	…	…
Investigations						
PCV performed	360 (98.6)	1901 (98.3)	0.80 (0.31‐2.06)	.644	…	…
Anemia	237 (65.8)	1514 (79.6)	2.03 (1.59‐2.59)	<.001	1.84 (1.38‐2.45)	<.001
Severe anemia	87 (24.2)	645 (33.9)	1.61 (1.24‐2.09)	<.001	…	…
Mean PCV, % (SD)	25.0 (10.1)	21.7 (9.7)	0.97 (0.96‐0.98)	<.001	…	…
Malaria test done	365 (100)	1934 (100)	Not applicable			
RDT	125 (34.3)	1101 (56.9)	2.54 (2.01‐3.21)	<.001	…	…
Microscopy	242 (66.3)	842 (43.5)	0.39 (0.31‐0.50)	<.001	…	…
Malaria test positive	365 (100)	1934 (100)	Not applicable			
RDT	125 (100)	1098 (99.7)	Not applicable			
Microscopy	240 (99.2)	838 (99.5)	1.75 (0.32‐9.59)	.521	…	…
HIV test done	166 (24.5)	780 (40.3)	0.81 (0.65‐1.01)	.067	…	…
HIV‐positive	4 (2.4)	3 (0.4)	0.16 (0.03‐0.71)	.016	…	…
CXR performed	2 (0.6)	10 (0.5)	0.94 (0.21‐4.32)	.940	…	…
CXR positive^c^	1/1 (100)	6/6 (100)	Not applicable			
Other admission diagnoses						
Pneumonia	25 (7.0)	53 (2.8)	0.42 (0.27‐0.66)	<.001	Not applicable	
Diarrhea	56 (15.6)	134 (6.9)	0.40 (0.29‐0.56)	<.001	Not applicable	
Sepsis	91 (25.4)	549 (28.4)	1.16 (0.90‐1.50)	<.251	Not applicable	
Seizures	134 (37.4)	449 (23.2)	0.51 (0.40‐0.64)	<.001	Not applicable	
Haemoglobinopathy	17 (4.8)	46 (2.4)	0.49 (0.28‐0.86)	.014	Not applicable	
Meningitis/encephalitis	30 (8.4)	161 (8.3)	0.99 (0.66‐1.49)	.972	Not applicable	
Severe acute malnutrition	13 (3.6)	24 (1.2)	0.33 (0.17‐0.66)	.002	Not applicable	
Asthma	0 (0.0)	0 (0.0)	Not applicable		Not applicable	
Treatment						
Parenteral antimalarial on day 1	302 (90.4)	1782 (92.4)	0.29 (0.87‐1.93)	.209	Not applicable	
IV fluids	363 (100)	1926 (99.8)	Not applicable		Not applicable	
Outcome						
Discharged well	304 (83.8)	1646 (85.6)	1.15 (0.85‐1.56)	.374	Not applicable	
Died	29 (8.0)	94 (4.9)	0.59 (0.38‐0.91)	.017	Not applicable	
Discharged against medical advice	24 (6.6)	142 (7.4)	1.13 (0.72‐1.76)	.605	Not applicable	
Transferred to other facility	6 (1.7)	42 (2.2)	1.33 (0.56‐3.15)	.520	Not applicable	
Missing data	2 (0.6)	10 (0.5)	0.94 (0.21‐4.32)	.940	Not applicable	

*Note*: Number (%) unless otherwise indicated. Anemia = PCV < 32%. Died = died in hospital or palliated at home. Feeding difficulty = unable to breastfeed or drink adequately. IQR = interquartile range, 25th to 50th centile. Severe anemia = PCV < 15%. Severe dehydration = sluggish capillary refill time (>3 s), sunken eyes, decreased skin turgor. Severe malaria = fever or history of fever AND positive malaria test AND at least one of the following: severe anemia; decreased conscious state; seizures. Severe respiratory distress = severe chest indrawing, grunting, gasping. Signs of shock = capillary refill < 3 s, weak pulse, low or unmeasurable blood pressure).

Abbreviations: PCV, packed cell volume (hematocrit); SD, standard deviation.

^a^ *P* values derived from univariate logistic regression.

^b^ Adjusted odds ratio derived from mixed effects logistic regression model that included random effects to adjust for clustering at the hospital level, and used stepwise regression to select variables that were independently associated with “detected” diagnosis at alpha .05 level (excluding treatment and outcome variables).

^c^ CXR denominator restricted to those with results available.

Antimalarial and intravenous fluid therapy were used frequently (90.6% and 99.6%), and univariate logistic regression found no association between children with missed versus detected severe malaria with regard to antimalarial therapy (OR, 0.29; 0.87‐1.93, *P* = .209) (Table 4). There was a negative association between mortality and detected severe malaria (OR, 0.59; 95% CI, 0.38‐0.91, *P* = .017).

### 3.5 Severe pneumonia and severe malaria

In total, 177 (1.2%) of children met WHO criteria for both severe malaria and severe pneumonia, representing 7.7% (177/2299) of those with severe malaria and 13.2% (177/1345) of those with severe pneumonia (Figure 1).

## 4 DISCUSSION

Pneumonia and malaria continue to be leading causes of mortality in children in sub‐Saharan Africa. Using routinely collected clinical data, this paper described children hospitalized with malaria and pneumonia syndromes in a malaria‐endemic low‐altitude region of southwest Nigeria. We identified “potential missed” severe pneumonia and malaria cases, and explored associations between “detected” diagnosis and participant demographic, clinical signs, and investigations, to give insight into current diagnostic practices.

We found that current diagnostic practices rely on clinical signs, pulse oximetry, and malaria testing (microscopy or rapid diagnostic testing, RDT), with little use of chest radiology. Almost half (557/1345, 41%) of children presenting with documented evidence of severe pneumonia (WHO case definition) were not given a pneumonia diagnosis. Compared with these “potential missed” diagnoses, children with “detected” severe pneumonia were four times more likely to receive parenteral antibiotics on day 1 of admission (OR, 4.03; 95% CI, 2.63‐6.16, *P* < .001), and showed a statistically nonsignificant but clinically significant lower odds of death (OR, 0.72; 0.51‐1.02, *P* = .067).

In contrast, only 16% (365/2299) of children presenting with evidence of severe malaria (WHO case definition) were not given a malaria diagnosis. While there was no difference in the likelihood of parenteral antimalarial administration between children with “potential missed” and “detected” severe malaria (OR, 0.29; 0.87‐1.93, *P* = .374), survival was better in those with a malaria diagnosis (OR, 0.59; 0.38‐0.91, *P* = .017). As a post hoc exploratory study, our findings should be interpreted with caution; however, they raise some interesting hypotheses.

### 4.1 Do some clinical signs and tests skew pneumonia diagnostic practices?

The case‐management approach to pneumonia, using clinical case definitions, has been extremely important in improving the diagnosis, treatment, and outcomes for children with pneumonia.^6^ However, the clinical signs of pneumonia (cough and difficult or fast breathing) are nonspecific and can overlap with other diseases that require different treatment (eg, HIV, tuberculosis, malaria).^6^ The challenge of overlapping diagnoses is particularly challenging in malaria‐endemic regions, where failure to identify pneumonia as a comorbid or alternate diagnoses may result in delayed or undertreatment.^5^, ^7^, ^20^, ^21^

We found that the majority (325/557, 58%) of children with “missed” severe pneumonia had signs of severe respiratory distress (240/557, 43.1%) or hypoxemia (149/472, 31.6%), suggesting substantial respiratory compromise. These results are consistent with other recent studies from large hospitals in Africa reporting similar hypoxemia prevalence in hospitalized children (aged <5 years) with pneumonia (28‐49%).^22^, ^23^, ^24^, ^25^, ^26^ We found that hypoxemia and severe respiratory distress were the strongest predictors of “detected” severe pneumonia (aOR 3.87; 2.58‐5.79, *P* < .001 | aOR 2.01; 0.37‐2.96, *P* < .001).

Conversely, positive malaria testing or detection of severe anemia were associated with lower likelihood of a pneumonia diagnosis (aOR 0.49; 0.34‐0.71, *P* < .001 | aOR 0.20; 0.09‐0.45, *P* < .001). These findings suggest that doctors had a lower index of suspicion for pneumonia in children that did not exhibit obvious respiratory signs, or who tested positive for other conditions. While this may be appropriate, it does raise the question of whether bedside testing for malaria and anemia contributed to missed pneumonia diagnoses. Importantly, the routine use of pulse oximetry in participating hospitals is atypical for many hospitals in low‐ and middle‐income countries, and diagnostic practices may be different where pulse oximetry is not used routinely.^14^, ^27^

### 4.2 Does lack of a pneumonia diagnosis compromise care and outcomes?

Without any “gold standard” testing for pneumonia (and low use of chest X‐ray radiography in participating hospitals), it is possible that some children included in our study had other primary diagnoses and were therefore not diagnosed with pneumonia. We found that two‐thirds (261/389, 67.1%) of children with WHO‐defined severe pneumonia were diagnosed with a different respiratory or systemic condition that may mimic severe pneumonia: sepsis (205, 37.8%); meningitis/encephalitis (28, 5.2%); or asthma (33, 6.1%).

Even if some of these cases could be explained by alternate diagnoses, in the absence of definitive testing, existing guidelines suggest that all children meeting WHO clinical criteria for severe pneumonia should receive treatment, including parenteral antibiotics.^4^, ^6^ Our findings show relatively high antibiotic coverage rates in both those with “detected” and “missed” severe pneumonia, suggesting that doctors recognized them as having a severe infection requiring antibiotics. However, there was a significant difference between those with “missed” severe pneumonia compared with those with “detected” severe pneumonia (85% vs 96%, OR, 4.03; 2.63‐6.16, *P* < .001). This suggests there is room for improvement in prompt antibiotic administration to all children meeting criteria for severe pneumonia, and raises questions about whether other standards of care for severe pneumonia may also have been lacking (eg, oxygen, saturation monitoring).

While we observed a statistically nonsignificant but clinically relevant association with lower mortality in those with “detected” vs “missed” severe pneumonia (OR, 0.72; 0.51‐1.02, *P* = .067), we cannot necessarily attribute this to diagnostic or treatment practices. The “missed” severe pneumonia subgroup were more likely to have neurological signs (eg, seizures, altered conscious state) and be given a diagnosis of seizures, meningitis, or severe acute malnutrition—all features that may be associated with poorer clinical outcomes. We face similar challenges interpreting the lower mortality in those with “detected” vs “missed” severe malaria.

### 4.3 What is the pathophysiology behind overlapping pneumonia and malaria syndromes?

While a large proportion of children diagnosed with pneumonia were given a co‐diagnosis of malaria (567/1799, 32%), overlap was not so obvious among those with WHO case definition severe pneumonia and severe malaria (177/1345, 13%, of severe pneumonia cases also met criteria for severe malaria). Previous studies have suggested that while clinical presentation of malaria and pneumonia may overlap, true severe disease overlap is uncommon, particularly in areas and times of lower malaria transmission.^5^, ^21^, ^28^, ^29^

The shared clinical features of severe pneumonia and severe malaria include: fever; tachypnoea and respiratory distress; altered conscious state. In severe pneumonia, respiratory distress is related to the degree of lung injury, the age of the child (younger children typically display more prominent respiratory signs), and the presence of comorbid conditions (eg, malnutrition, anemia). Altered conscious state typically reflects impaired cerebral oxygenation, related to hypoxemia and/or impaired perfusion.

In severe malaria, respiratory distress is a relatively common finding and is a well‐established predictor of mortality among children with severe malaria, increasing their risk of death approximately fourfold.^30^, ^31^, ^32^ We observed a relatively high prevalence of hypoxemia (261/1854, 14%) and severe respiratory distress (217/2299, 9%) among children meeting WHO criteria for severe malaria. Data from the multicentre AQUAMAT trial showed that 17% of children hospitalized with severe malaria had signs of respiratory distress,^32^ while smaller studies have reported variable incidence between 14% and 60%.^31^, ^33^

In malaria, compensatory hyperventilation related to metabolic acidosis is the most common driver of respiratory signs—causing tachypnoea, chest indrawing, nasal flaring, and deep breathing—and is probably the primary mechanism for excess mortality.^30^ Severe anemia may similarly result in increased respiratory effort, particularly if associated with cardiac failure. While anemia‐associated cardiac failure is anecdotally common, limited data from one Kenyan study reported mild to moderate cardiomegaly in 11% (7/64) of children with malaria and severe anemia but no evidence of pulmonary edema or congestion.^5^ Similar to previous studies, we found very high incidence of severe anemia among children with severe malaria (732/2261, 32%).^5^, ^28^ We did not collect data on cardiac failure.

Adult studies suggest that acute respiratory distress syndrome (ARDS) is relatively common in severe malaria, noting that respiratory symptoms may develop following treatment when malaria parasite blood levels have dropped.^34^, ^35^ This suggests that parasitic infiltration into the lungs may not be the only cause of respiratory signs, and that posttreatment inflammatory effects (perhaps immune‐mediated) may be major contributors.^34^, ^35^ However, ARDS is less common in children with severe malaria and it is unclear how much parasitic sequestration in child lungs contributes to respiratory signs.^35^

### 4.4 How can frontline healthcare workers disentangle severe pneumonia and malaria?

Previous studies in similar contexts have failed to find clinical or laboratory signs that reliably distinguish between severe malaria and pneumonia.^5^, ^28^ In Mozambique, younger age, dehydration, lower PCV counts, and hyperbilirubinaemia were predictive of severe malaria, while preadmission antibiotic treatment, crackles, unilateral chest signs, nasal flaring, hypoxemia, poorer nutritional status, and higher leukocyte counts were predictive of severe pneumonia.^28^ In Kenya, severe indrawing, unilateral chest signs, and hypoxemia were found to be specific predictors of radiographic pulmonary consolidation, but only crackles had reasonable specificity and sensitivity.^5^ Authors of the papers from Mozambique and Kenya emphasized the inability of any combination of signs to reliably distinguish between severe pneumonia and malaria, and recommended that all hospitalized children meeting criteria for severe pneumonia and malaria be treated for both—especially in the absence of chest X‐ray radiography facilities.^5^, ^28^

Our findings suggest that doctors were using signs of severe respiratory distress and hypoxemia to diagnose pneumonia appropriately. However, doctors may have been overly reliant on these insensitive respiratory signs and unduly influenced away from a pneumonia diagnosis when other signs were present that could still be consistent with severe pneumonia (eg, diarrhea, altered conscious state, feeding difficulties)—or when bedside testing suggested an additional diagnosis (eg, malaria, anemia). While the proportion of “missed” severe malaria diagnoses was relatively low, our results suggest that doctors appropriately used severe anemia as a predictor of malaria diagnosis but may have been unduly swayed away from a malaria diagnosis by other signs that could still be consistent with severe malaria (eg, diarrhea, severe dehydration).

### 4.5 Limitations

This study involved post hoc exploratory data analysis and is therefore primarily hypothesis generating. We relied on documented clinical information and had minimal missing data, thanks to the close attention of our research nurses at each site. A pediatrician assessed the level of detail in case note documentation before commencing data collection, but we did not independently verify the documented clinical signs or diagnostic results. Where clinical signs were not documented, we assumed that they were not present; however, this may have resulted in an underestimate of signs. We focussed comparative analysis on those meeting WHO definitions of severe pneumonia and malaria to exclude those with mild respiratory disease or incidental parasitaemia. We were limited by the lack of independent confirmatory testings, such as routine chest X‐ray radiography or blood cultures, which could have more accurately defined severe pneumonia and invasive bacterial disease.

While we did not exclude children with HIV from analysis, this study was conducted in an area with low HIV‐prevalence and results may not be generalizable to contexts with higher HIV prevalence. Southwest Nigeria is relatively wealthier and better served with health services and staff than other regions of Nigeria (and sub‐Saharan Africa more broadly), and participating hospitals had received particular assistance with pulse oximetry and oxygen therapy. Diagnostic practices may be different in other areas of Nigeria and Africa that have lower socioeconomic indices, fewer healthcare workers, and less available hospital services. Routine use of pulse oximetry by nurses in our study is relatively unusual for hospitals in sub‐Saharan Africa,^27^ and may have influenced diagnostic treatment practices.^14^

## 5 CONCLUSIONS

Clinical overlap between children presenting with malaria and pneumonia continues to present diagnostic challenge to healthcare workers in Nigerian hospitals. Current diagnostic practices miss up to half of severe pneumonia cases and may result in delayed or undertreatment and poorer clinical outcomes. Severe malaria diagnoses are less frequently missed, and antimalarial treatment coverage is high. Healthcare workers should be encouraged to consider pneumonia in all severely unwell children with respiratory signs, even if they are also treated for malaria or another condition.

## CONFLICT OF INTERESTS

HG, AAB, AIA, EN, TD, and AGF received payment for services on this project from the funder (Bill and Melinda Gates Foundation, OPP1123577). The other authors declare that there are no conflict of interests.

## AUTHOR CONTRIBUTIONS

HG, AIA, AGF, and TD conceived and designed the study. HG, AAB, AIA, and AGF participated in project implementation. AAB and OBO coordinated data collection and database entry. HG did the data analysis. HG, AAB, AG, EN, SQ, TD, and AGF contributed to the interpretation of results. HG drafted the manuscript. AAB, AG, EN, SQ, TD, and AGF provided substantial comments to the writing of the manuscript. All the authors read and approved the final manuscript.

## ETHICS STATEMENT

This study obtained ethics approval from the University of Melbourne (1543797.1) and the University of Ibadan/University College Hospital Ethics Committee, Ibadan, Nigeria (UI/EC/16/0413).
